# Concomitant depletion of PTEN and p27 and overexpression of cyclin D1 may predict a worse prognosis for patients with post-operative stage II and III colorectal cancer

**DOI:** 10.3892/ol.2014.2350

**Published:** 2014-07-14

**Authors:** JING LI, LIN-LIN YIN, KE-LI SU, GANG-FENG ZHANG, JING WANG

**Affiliations:** Department of Oncology, The Fourth People’s Hospital of Jinan, Jinan, Shandong 250031, P.R. China

**Keywords:** colorectal cancer, prognosis, PTEN, p27, cyclin D1

## Abstract

Prognostic markers for colorectal cancer (CRC) have not yet been fully investigated. Phosphatase and tensin homolog (PTEN), p27 and Cyclin D1 play significant roles in tumorigenesis and cell cycle regulation, and therefore require evaluation for their prognostic value in this disease. The aim of the present study was to assess the prognostic value of the single and combined expression of PTEN, p27 and Cyclin D1 in CRC patients. Protein expression levels of PTEN, p27 and Cyclin D1 were examined by immunohistochemistry from 61 patients with CRC in either stage II or III. In the CRC tissues, the frequencies of PTEN(−), p27(−) and Cyclin D1(+) expression were 42.62% (26/61), 32.79% (20/61) and 45.90% (28/61), respectively. Depletion of PTEN and p27 was more common with respect to stage III, low grade and lymph node metastasis compared with stage II, moderate grade and no lymph node metastasis (P<0.05). Cyclin D1-positive expression was frequently detected in CRC of stage III, with lymph node metastasis and deeper invasion (P<0.05). The depletion of PTEN was significantly correlated with the loss of p27 (P<0.001) and with the increased expression of Cyclin D1 (P<0.001). PTEN(−) and/or p27(−) expression was significantly correlated with Cyclin D1(+) expression (P<0.05). Combined PTEN(−)/p27(−)/Cyclin D1(+) expression was correlated with a significant decrease in overall survival time (P<0.05). Combined p27(−) and Cyclin D1(+) expression indicated a worse overall survival time than other combined expression patterns. These findings indicate that the single expression of PTEN(−), p27(−) and Cyclin D1(+) and the combined detection of p27(−) and Cyclin D1(+) may be used as prognostic markers for overall survival time in CRC.

## Introduction

The incidence of colorectal cancer (CRC) has been increasing over the past few decades, resulting in the disease being the fourth most common cancer in Asia (1). Although treatments with radical surgery, radiotherapy and systemic chemotherapy are performed, the clinical outcomes of CRC remain unsatisfactory. Adjuvant chemotherapy is recommended universally for all patients with stage III CRC, but the role of adjuvant chemotherapy in CRC patients at stage II has not yet been well established (2). Only 3.6% of stage II patients benefit from adjuvant chemotherapy; >80% of stage II patients are cured only by surgery (3,4). At present, although numerous prognostic markers, such as SMAD4 and CD44v6 (5,6), have been investigated, only a few are clinically applied due to the controversial value of prognostic factors. Furthermore, the high expenses amassed in detecting potential prognostic markers prevent them from being widely used clinically. Therefore, it is imperative to identify the prognostic biomarkers that can be assessed by low-expense methods widely used in clinics and screen for the CRC subgroup that can benefit from adjuvant chemotherapy to avoid unnecessary treatment with chemotherapy.

Aberrations in cell cycle regulatory proteins are common in a number of tumors (7,8). The cell cycle is a highly organized process regulated by cyclins, cyclin-dependent kinases (CDKs) and CDK inhibitors (CKIs). Overexpression of cyclins, CDKs or loss of CKIs, which can promote the procession of cell cycle, may contribute to uncontrolled proliferation (9). The cell cycle comprises of four phases, the G_1_, S, G_2_ and M phases. G_1_/S, as one of various important checkpoints, plays a critical role in controlling cell cycle progression. Aberrations of proteins regulating G_1_/S checkpoints may be involved in tumorigenesis (10,11). Numerous molecules are associated with regulating the cell cycle transition from the G_1_ to the S phase. Of these regulators, the tumor suppressor phosphatase and tensin homolog (PTEN) and p27 (CDKI 1B, P27KIP-1) have a negative role, whereas Cyclin D1 acts as a positive regulator (12,13).

The phosphatidylinositol 3-kinase (PI3K) signaling pathway is a significant pathway regulating cell growth, survival and proliferation (14). PTEN acts as a key negative regulator of the PI3/Akt pathway (15) by dephosphorylating phosphoinositol-3,4,5-triphosphate (PIP3), preventing Akt activation and downregulating the pathway. Depletion of PTEN has been reported to accelerate cell proliferation (12) and promote the G_0_ to G_1_ cell cycle transition. Loss of PTEN expression has been linked to a worse prognoses in patients with CRC (16). However Ghiţă *et al* (17) demonstrated that PTEN showed no statistically prognostic value in CRC.

p27 belongs to the CKI family and plays a critical role in inhibiting the transition from the G_1_ to S phase by binding and inhibiting cyclin/CDKs (13). High expression levels of p27 are present in the G_0_/G_1_ phase in the normal cell cycle. Following mitogenic stimulation, rapid degradation of p27 occurs, allowing the promotion of cell proliferation via the action of CDK2/cyclin. Thus, depletion of p27 may contribute to the uncontrolled proliferation of malignant cells. A previous study (17) showed that the decreased expression of p27 was associated with a poor prognosis in CRC. Al-Maghrabi *et al* (18) showed that p27 positive expression was associated with a high recurrence rate, in conflict with the findings by Zlobec *et al* (19).

Cyclin D1 is an important cyclin that binds partners cyclin-dependent kinase (CDK)4 and CDK6 and forms active complexes that promote G_1_- to S-phase progression by phosphorylating and inactivating the retinoblastoma (Rb) protein (20). The ability of Cyclin D1 to drive the cell cycle forward can be inhibited by CKIs, such as p21 and p27 (21). Increased levels of Cyclin D1 may be associated with a shorter survival time (22). However, Belt *et al* (23) showed that Cyclin D1-positive staining was associated with a good prognosis. The prognostic significance of Cyclin D1 in CRC has yet to be resolved.

PTEN co-ordinates G_1_-phase arrest, upregulating p27 through its lipid phosphatase activity and downregulating Cyclin D1 through its phosphatase activity (24). These three proteins interact as a network.

At present, none of these three proteins has been clinically applied. The association of the combined detection of PTEN/p27, PTEN/Cyclin D1 and p27/Cyclin D1 with survival in CRC has not been documented. The prognostic value of PTEN, p27 and Cyclin D1 should also be reevaluated. Whether joint examination of PTEN, p27 and Cyclin D1 may predict the clinical outcome for CRC patients requires investigation. The aim of the present study was to use immunohistochemistry to determine the expression patterns of PTEN, p27 and Cyclin D1 in normal and tumor epithelium in stage II/III CRC patients. The correlation of the expression of the three proteins with the clinicopathological characteristics and overall survival time was analyzed respectively. The correlation between concomitant expression of these markers and overall survival time was assessed, and an analysis of whether combined detection can provide additional prognostic clues in order to contribute to identifying precise prognostic biomarkers was performed.

## Materials and methods

### Materials

The study group was comprised of 61 patients with CRC in either stage II or III who underwent surgery between March 2005 and May 2006 at The Fourth People’s Hospital of Jinan (Jinan, Shandong, China). A total of 32 patients were at stage II and 29 at stage III, according to the 7th American Joint Committee on Cancer staging system (25). Another 20 samples of corresponding adjacent non-cancerous tissues were used as controls. All tissues were collected from the Pathology Department (The Fourth People’s Hospital of Jinan), and all patients were followed up until May 31, 2011. Tissue samples were fixed in 10% formalin and embedded in paraffin. Of the 61 cases, 38 were male and 23 were female. The mean age at diagnosis was 64 years (range, 27–85 years). A total of 46 cases were of moderate grade and 15 were of low grade, while 29 had pathological lymph node metastasis and 32 did not. The number of pT3 and pT4 cases was 31 and 32, respectively. All cases were followed up for overall survival analysis subsequent to being diagnosed until May 31, 2011. The follow-up period was initiated on the date when the patient was first diagnosed with CRC and lasted until mortality or the end of follow-up. The median follow-up duration was 6.7 years. Patients provided written informed consent and the study was approved by the ethics committee of the Fourth People’s Hospital of Jinan.

### Immunohistochemical staining

Immunohistochemical studies for PTEN, p27 and Cyclin D1 were performed on formalin-fixed, paraffin-embedded surgical sections, consisting of the 61 CRC and 20 normal tissues. The tissue sections were deparaffinized and soaked in 0.01 M sodium citrate buffer, and the cell antigens were retrieved. Subsequent to blocking non-specific binding with 10% bovine serum, the sections were incubated with anti-human mouse monoclonal antibodies against PTEN (1:100), p27 (1:100) and cyclin D1 (1:100) (all Santa Cruz Biotechnology, Inc., Santa Cruz, CA, USA) overnight at 4°C. Anti-rabbit goat polyclonal antibodies (Vector Laboratories, Burlingame, CA, USA) were used at dilutions of 1:50. The bound antibodies were visualized using the avidin-biotin-peroxidase method (Vector Laboratories). All sections were counterstained with hematoxylin. Negative control sections were prepared using Tris-buffered saline instead of primary antibody. Sections of tonsil tissue obtained from the excision of the tonsils with known PTEN expression were used as positive controls for PTEN.

### Criteria for judging

The samples were assessed in a blind manner by two investigators with no knowledge of the clinicopathological data. Five microscopic fields were randomly viewed to calculate the mean number of positive cells. The staining was evaluated only in the areas with well-preserved tissue morphology and away from necrosis or artefacts. PTEN expression was observed in the cytoplasm. The intensity was scored according to a four-tier system, as follows: 0, no staining; 1, weak; 2, moderate; and 3, strong. Another 1, 2, or 3 points was assigned if the percentage of positive cells was <25, 25–50, or >50%, respectively. Specimens were defined as positive if the score was ≥4 (12).

Staining for p27 and Cyclin D1 was found in the nucleus. All cases were scored as either positive (≥10% of tumor cells with strong nuclear staining), negative (<10% of tumor cells with strong nuclear staining) or non-informative (26). A staining extent of >5% of the tumor cells was considered positive for Cyclin D1, while ≤5% was considered negative (22).

### Statistical analysis

Fisher’s exact test was adopted to compare the difference in expression levels of the three proteins between the CRC and control groups. The χ^2^ test was used to examine the association between PTEN, p27 and Cyclin D1 expression and the clinicopathological parameters. Spearman’s linear regression test was used to evaluate correlations among protein expression levels. Survival curves were created using the Kaplan-Meier method, and any differences in the survival curves were compared by the log-rank test. P<0.05 was considered to indicate a statistically significant difference. All statistical analyses were performed with SPSS 13.0 statistical software (SPSS, Inc., Chicago, IL, USA).

## Results

### Immunohistochemical expression of PTEN, p27 and Cyclin D1

**Figure 1:**
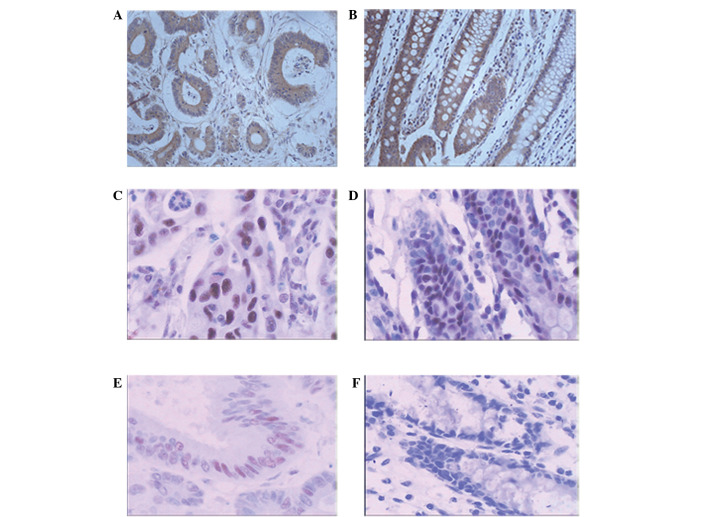
Immunohistochemistry for PTEN, p27 and Cyclin D1 in CRC tissues and normal colonic mucosa adjacent to cancer tissues. Representative slides demonstrating immunohistochemical staining of CRC and adjacent normal mucosa. (A) Expression of PTEN is strongly detected in the cytoplasm of the CRC cells, but not in the nucleus. (B) PTEN expression is also observed in the matched normal colonic tissues. (C) The tumor cells exhibit diffuse nuclear staining for p27. (D) Strong nuclear staining is present in the matched normal colonic tissues. (E) Cyclin D1 immunoreactivity is confined to the nucleus in the CRC tissues. (F) No staining is present in the normal matched colonic tissues. All images are captured at ×40 magnification. PTEN, phosphatase and tensin homolog; CRC, colorectal cancer.

Positive staining for PTEN was detected in the cytoplasm, but not in the nucleus. Of the 61 tumors analyzed, 26 (42.62%) demonstrated loss of PTEN expression, while 35 (57.38%) retained PTEN expression (Fig. 1A). All 20 (100%) in the control group tested positive (Fig. 1B). A total of 20 out of 61 (32.79%) cases of CRC showed depleted p27 expression, and 41 out of 61 (67.21%) cases revealed p27 nuclear accumulation (Fig. 1C). However, all 20 (100%) showed nuclear positivity in the control group (Fig. 1D). Cyclin D1 immunoreactivity was confined to the nucleus. The positive expression of Cyclin D1 was observed in 28 out of 61 (45.90%) cases of CRC (Fig. 1E), while no staining was observed in the control group (Fig. 1F). Expression levels of PTEN, p27 and Cyclin D1 between the CRC and control groups were statistically different (P<0.05) (Table I).

### Correlation of PTEN, p27 and Cyclin D1 expression with the clinicopathological parameters

Table II shows that the expression levels of PTEN and p27 were correlated with tumor stage, histological grade and lymph node metastasis, and not with gender, age or depth of tumor invasion. The depletion of PTEN and p27 were more common in cases of stage III, low grade and lymph node metastasis compared with those of stage II, moderate grade and no lymph node metastasis (P<0.05, Table II). Similarly, the expression of Cyclin D1 showed significant correlation with tumor stage, lymph node metastasis and depth of tumor invasion, and no correlation with gender, tumor grade and age. Cyclin D1-positive expression was frequently detected in CRC of stage III, with lymph node metastasis and deeper invasion (P<0.05, Table II).

### Correlation among PTEN, p27 and Cyclin D1 expression

The depletion of PTEN expression was significantly correlated with the loss of p27 (r=0.810; P<0.001) and with the increased expression of Cyclin D1 (r=−0.470; P<0.001). However, the decreased expression of p27 was inversely associated with the Cyclin D1 level (r=−0.548; P<0.001).

### Correlation of expression PTEN, p27 and Cyclin D1 with prognosis

**Figure 2:**
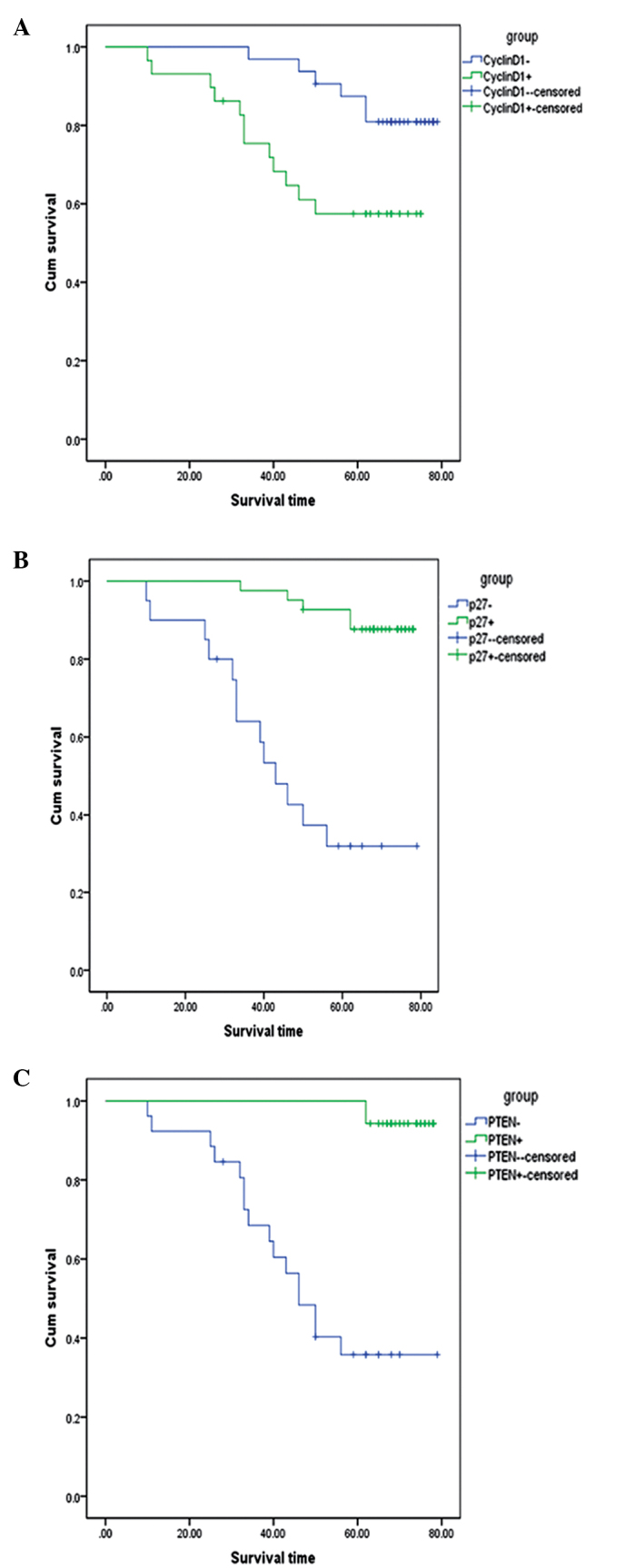
Overall survival curves for patients with CRC based on the level of PTEN, p27 and Cyclin D1 expression. (A) The overall survival time for the patients with PTEN-positive expression was higher than that for the patients with PTEN depletion (χ^2^=28.71; P<0.001). (B) The patients who expressed high levels of p27 survived longer than those who demonstrated p27 depletion (χ^2^=26.88; P<0.001). (C) The patients with Cyclin D1-positive expression exhibited shorter survival times compared with those with no staining for Cyclin D1 (χ^2^=5.36; P=0.021). Analyses were performed using the Kaplan-Meier method. PTEN, phosphatase and tensin homolog; CRC, colorectal cancer.

Of the 61 patients, 35 patients showed PTEN-positive staining, with a mean survival time of 77.1 months, whereas PTEN depletion was found in 26 patients, who exhibited a mean survival time of 51.5 months (χ^2^=28.71; P<0.001) (Fig. 2A). A total of 41 patients expressed high p27 levels, and 20 patients demonstrated p27 depletion, with a mean survival rate of 74.7 and 48.7 months, respectively (χ^2^=26.88 ; P<0.001) (Fig. 2B). In total, 29 patients showed Cyclin D1-positive expression and 32 exhibited no staining, with a mean survival time of 56.9 and 73.8 months, respectively (χ^2^=5.36; P=0.021) (Fig. 2C).

**Figure 3:**
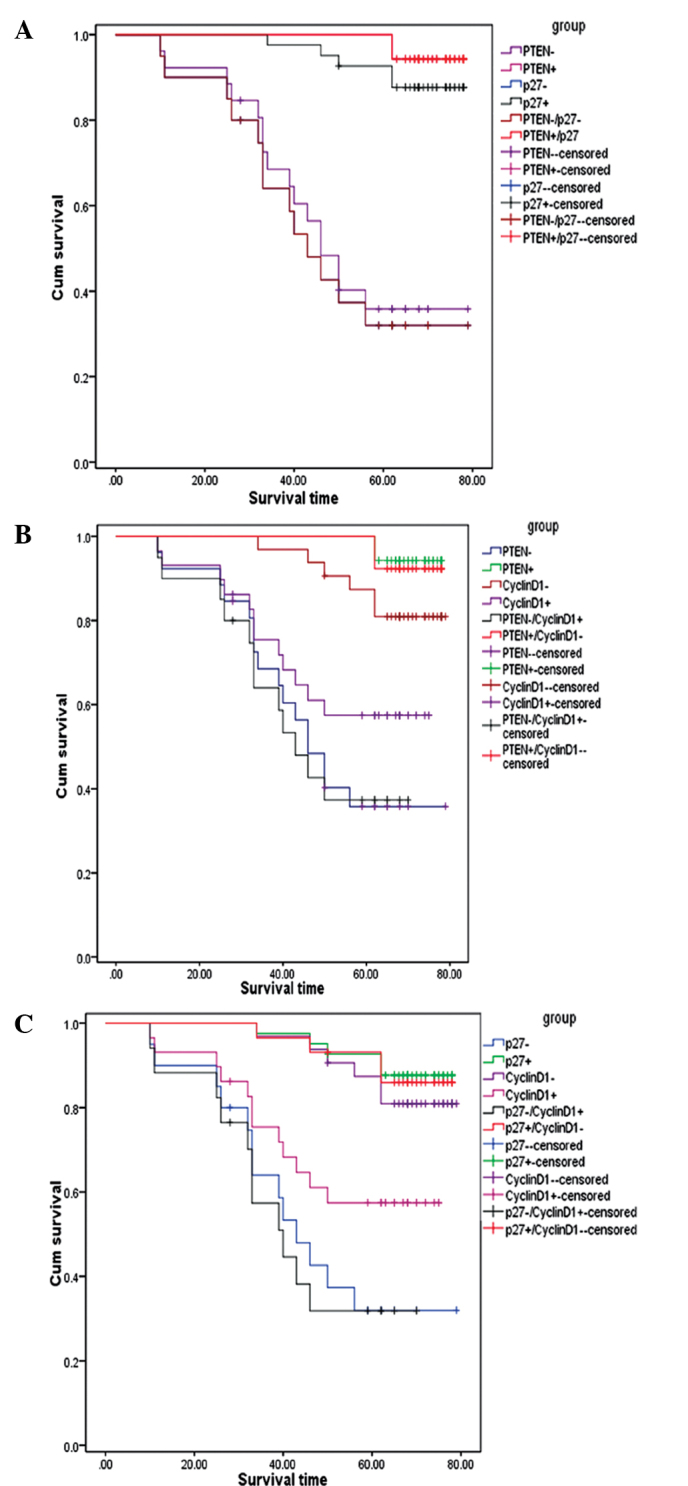
Overall survival curves for CRC patients based on combined detection of PTEN/p27, PTEN/Cyclin D1 and p27/Cyclin D1 expression. (A) Patients with PTEN(+), PTEN(−), p27(+), p27(−), PTEN(+)/p27(+), and PTEN(−)/p27(−) exhibited a mean survival time of 77.1, 51.5, 74.7, 48.7, 77.1 and 48.7 months, respectively. Patients with PTEN(−)/p27(−) expression exhibited the worst survival times. (B) Patients with PTEN(+), PTEN(−), Cyclin D1(−), Cyclin D1(+), PTEN(+)/Cyclin D1(−) and PTEN(−)/Cyclin D1(+) expression had a mean survival time of 77.1, 51.5, 73.8, 56.9, 76.8 and 46.6 months, respectively. Patients with PTEN(+) expression exhibited the best survival times, while joint examination of PTEN(+)/Cyclin D1(−) expression did not provide extra prognostic information. However, combinations of PTEN(−)/Cyclin D1(+) expression provided a more adverse prognosis compared with detection of PTEN(−) and Cyclin D1(+) expression alone. (C) Patients with p27(+), p27(−), Cyclin D1(−), Cyclin D1(+), p27(+)/Cyclin D1(−) and p27(−)/Cyclin D1(+) expression had a mean survival time of 74.7, 48.7, 73.8, 56.9, 74.2 and 43.5 months, respectively. Combination of p27(+)/Cyclin D1(−) expression did not provide additional predictive information for clinical outcome compared with detection of p27(+) and Cyclin D1(−) expression alone. Notably, patients with p27(−)/Cyclin D1(+) expression had worse survival times. PTEN, phosphatase and tensin homolog; CRC, colorectal cancer.

To determine whether joint examination of these proteins could provide additional information for prognosis, the survival Kaplan-Meier test for the combination of PTEN/p27, PTEN/Cyclin D1 and p27/Cyclin D1 was analyzed. The results indicated that PTEN and p27 are markers for good prognosis, whereas Cyclin D1 predicts adverse prognosis. In Fig. 3A, the survival curves among the patients with PTEN(+), PTEN(−), p27(+), p27(−), PTEN(+)/p27(+) and PTEN(−)/p27(−) expression was assessed. The patients with PTEN(+), PTEN(−), p27(+), p27(−), PTEN(+)/p27(+) and PTEN(−)/p27(−) expression exhibited a mean survival time of 77.1, 51.5, 74.7, 48.7, 77.1, and 48.7 months, respectively. The patients with PTEN(+)/p27(+) expression exhibited a 2.4-months longer survival time than the patients with p27(+) expression alone. Notably, the patients with PTEN(−)/p27(−) expression exhibited a 2.8-months shorter survival time compared with those with PTEN(−) expression alone. As expected, the patients with PTEN(+)/p27(+) expression exhibited the best overall survival time, whereas the patients with PTEN(−)/p27(−) expression exhibited the worst survival time.

Fig. 3B shows that the patients with PTEN(+), PTEN(−), Cyclin D1(−), Cyclin D1(+), PTEN(+)/Cyclin D1(−) and PTEN(−)/Cyclin D1(+) expression had a mean survival time of 77.1, 51.5, 73.8, 56.9, 76.8 and 46.6 months, respectively. Evidently, those patients with PTEN(+), Cyclin D1(−) and PTEN(+)/Cyclin D1(−) expression had improved clinical outcomes. The patients with PTEN(+) expression showed the best survival times, while the joint examination of PTEN(+)/Cyclin D1(−) expression did not provide extra prognostic information. However, combinations of PTEN(−)/Cyclin D1(+) expression provided a more adverse prognosis compared with detection of PTEN(−) and Cyclin D1(+) expression alone. Patients with PTEN(−)/Cyclin D1(+) expression exhibited a 4.9- and 10.3-months shorter survival time compared with those with PTEN(−) and Cyclin D1(+) expression alone, respectively.

Fig. 3C shows that the patients with p27(+), p27(−), Cyclin D1(−), Cyclin D1(+), p27(+)/Cyclin D1(−) and p27(−)/Cyclin D1(+) expression had a mean survival time of 74.7, 48.7, 73.8, 56.9, 74.2 and 43.5 months, respectively. It was observed that the patients with p27(+)/Cyclin D1(−) expression survived only 0.4 months longer than those with Cyclin D1(−) expression and 0.5 months less than those with p27(+) expression alone. Thus, the combination of p27(+)/Cyclin D1(−) expression did not provide additional predictive information to the clinical outcome compared with detection of p27(+) and Cyclin D1(−) expression alone. However, the study indicated that the expression of p27(−), PTEN(+) and p27(−)/Cyclin D1(+) were associated with a poor survival time. Notably, patients with p27(−)/Cyclin D1(+) had worse survival. Those patients with p27(−)/Cyclin D1(+) expression had mean survival times of 5.2 and 13.4 months in contrast to those with p27(−) and Cyclin D1(+) alone, respectively.

## Discussion

The gene product of PTEN, lipid phosphatase, can dephosphorylate PIP3 (15). The lipid phosphatase can inhibit PI3K activity, which is normally essential for activation of protein kinase B, a serine/threonine kinase involved in cell growth and survival (15). PTEN can also induce G_1_-phase cell cycle arrest by negatively regulating the PI3K/Akt signaling pathway (27). Loss of PTEN results in increased Akt activity and uncontrolled cell proliferation. Depletion of PTEN has been associated with a large number of human cancers, including bladder cancer, CRC and breast cancer (12,19,28). In the present study, PTEN was frequently deleted in CRC of poor grade, with lymph metastasis and high clinical stage, indicating the involvement of PTEN deletion in colorectal carcinogenesis. At present, the prognostic value of PTEN has not been documented in CRC. Jin *et al* (16) reported significantly higher three- and five-year survival rates in PTEN protein-positive patients than in PTEN protein-negative patients. However, in a multivariate analysis, Hsu *et al* (29) demonstrated that PTEN expression was not of independent prognostic value in CRC. In the present study, it was observed that patients with PTEN-positive expression exhibited improved prognoses compared with those with PTEN-negative staining, which was in line with the study by Jin *et al* (16).

p27 is a member of the cip1/kip1 family of CDKIs that regulate the progression of cells from late G1-phase into the S-phase (30). Loss of p27 may contribute to the uncontrolled proliferation of malignant cells. It is known that p27 expression is lower in a series of human cancers, including CRC and breast and lung cancer (20,31,32). In the present study, 32.79% of tumors showed lower or depleted p27 expression, in agreement with the report of Al-Maghrabi *et al* (18). The present study found that there were no significant correlations between the loss of p27 expression and gender, age and depth of invasion. Depletion of p27 revealed highly significant correlation with tumor grade, lymph metastasis and clinical stage. This observation is in agreement with the study by Shapira *et al* (33), who reported low p27 levels associated with poorly-differentiated tumors, but not with age, gender and clinical stage in CRC. However, Al-Maghrabi *et al* (18) showed that p27 expression was only associated with depth of invasion and not with tumor grade, lymph metastasis and clinical stage in CRC. Although p27 may be significantly associated with tumor invasiveness characteristics, including grade, depth of invasion and clinical stage, the prognostic value of p27 remains controversial. Al-Maghrabi *et al* (18) reported that colorectal tumors with increased p27 expression showed a higher recurrence rate and shorter disease-free patient survival time than those with lower level or completely depleted p27 expression. However, in the present study, it was observed that patients with p27 expression had longer survival times than those with no p27 expression, which was consistent with the study by Bertagnolli *et al* (26), which described that loss of p27 as associated with reduced survival in colon cancer patients. Differences in the technical aspects of recording p27 expression or in the interpretation of its expression may result in these apparently discrepant findings.

Cyclin D1 is an important regulator of G_1_ to S-phase progression. Together with its binding partners, CDK4 and CDK6, Cyclin D1 forms active complexes that promote cell cycle progression by phosphorylating and inactivating the Rb protein (20). Defective regulation of this important checkpoint may result in uncontrolled cellular proliferation. The overexpression of Cyclin D1 has been found in a variety of tumors types, including breast, oral and oropharyngeal squamous cell carcinoma and CRC (13,34,35). In the present study, increased expression of Cyclin D1 was observed, in line with the findings of Ioachim (35). However, the expression of Cyclin D1 and its correlation with the clinicopathological parameters remain unclear. Ioachim (35) found that the overexpression of Cyclin D1 was significantly associated with tumor stage and lymph node involvement in CRC. Meanwhile, Mao *et al* (22) found no association between Cyclin D1 expression and gender, age, depth of invasion, tumor differentiation, clinical stage and lymph node metastasis in colonic adenocarcinoma. In the present study, it was observed that increased Cyclin D1 expression was correlated with advanced clinical stage, lymph node metastasis and deeper tumor invasion depth, but not with age, gender or histological grade. These results indicate that Cyclin D1 may be associated with the more aggressive phenotype of CRC. The association between Cyclin D1 expression and overall survival is controversial. A previous study found that Cyclin D1-positive expression was associated with shorter survival times in patients with CRC, but that it was not an independent predictor of survival (22). However, according to the study by Hwang *et al* (36), the overexpression of Cyclin D1was associated with improved outcomes in breast cancer. In the present study, it was found that patients with Cyclin D1-positive expression had a poorer prognosis, in contrast to the findings of Hwang *et al* (36) and in line with those of Mao *et al* (22).

PTEN, p27 and Cyclin D1 belong to the cell cycle regulatory proteins. PTEN and p27 play a significant role in regulating the transition from the G_1_ phase to the S phase. PTEN negatively mediates p27 activation and inhibits the binding of Cyclin D1 with CDK. Depletion of PTEN contributes to deactivating p27 through the suppression of AFX/FKHR (37). Similarly, loss of PTEN can activate pAKT, which may activate Cyclin D1 through mTOR (37). A previous study observed that p27 and Cyclin D1 may be the crucial downstream targets of the PTEN-mediated pathway. Thus, concomitant expression of PTEN and p27 exhibit a synergistic role in the progression from G_1_ to S phase. PTEN expression has been positively correlated with p27 (31). The present study also observed a positive correlation between PTEN and p27 expression, in line with the study by Tsutsui *et al* (31). Certain studies have evaluated the prognostic value of the combination of PTEN and p27 protein expression in breast cancer and prostate cancer. The combined loss of PTEN and p27 expression was associated with an aggressive phenotype and a poor prognosis in breast and prostate carcinomas (31,38). However, the joint detection of PTEN and p27 in CRC has not been documented. In the present study, patients with joint loss of PTEN and p27 had worse survival times compared with those with PTEN and p27 loss alone. Patients with the combined deletion of PTEN and p27 had a 2.8-months shorter survival time than those with PTEN deletion alone. These results indicate that a combined loss of PTEN and p27 function strongly promotes the progression of CRC. These findings are consistent with the results of the studies on breast and prostate carcinoma (31,38).

There was, however, no definite association between PTEN and Cyclin D1. In breast cancer studies, statistically significant associations have been found between PTEN and cyclin D1 expression patterns (34). However, Fiano *et al* (39) observed no direct correlation between Cyclin D1 overexpression and the loss of PTEN in CRC. In the present study, an inverse association between Cyclin D1 and loss of PTEN was observed. The prognostic value of the combined examination of PTEN and Cyclin D1 in CRC has not been documented. In the present study, patients with the combined detection of Cyclin D1(+)/PTEN, PTEN(−) and CylinD1(+) exhibited survival times of 46.6, 51.5 and 56.9 months, respectively. Joint detection provided a worse prognostic value in CRC, indicating that loss of PTEN exhibited a synergistic role with Cyclin D1-positive expression in tumor progression.

p27 interacts with Cyclin D1 in the progression of the cell cycle. As a potential inhibitor of the cyclin-CDK complex, p27 can negatively regulate the ability of the complex to phosphorylate other proteins like pRb, preventing Cyclin D1-CDK4 from binding. Thus, the decreased expression of p27 has been correlated with Cyclin D1 overexpression. However, according to the findings of Engin *et al* (34) in breast cancer, p27 expression was not associated with Cyclin D1. With regard to CRC, Ioachim (35) reported that overexpression of Cyclin D1 was not associated with p27. Ye *et al* (40) found that the expression of Cyclin D1 observably increased, while p27 expression markedly decreased *in vitro* in gastric cancer cells. In the present study, p27 was inversely correlated with Cyclin D1. At present, there are no reports stating that the concomitant detection of p27 and Cyclin D1 is associated with the prognosis of CRC. In the present study, it was observed that the coexistence of p27-negative and Cyclin D1-positive staining showed worse patient survival times compared with p27-negative and Cyclin D1-positive expression alone (43.5 vs. 48.7 vs. 56.9 months). Patients in the combined group had a 13.4-months shorter survival time than those with Cyclin D1-positive expression, indicating that p27 may coordinate with Cyclin D1 in the progression of colorectal carcinogenesis.

In conclusion, the findings of the present study confirmed the prognostic value of PTEN, p27 and Cyclin D1 in CRC. Furthermore, PTEN was found to positively correlate with p27 and negatively correlate with Cyclin D1, indicating the combined regulation of these cell cycle checkpoints during the process of neoplastic progression. Moreover, joint examination can provide more adverse prognostic clues to those patients with a poor prognosis, not improved prognostic indexes to those with a good prognosis. These joint examinations may assist in the selection of patients with a worse prognosis; these patients can then be directed to the more intensive treatment of adjuvant chemotherapy. Thus, these selections may enable more accurate subgrouping of patients in terms of potential benefit from adjuvant therapy to avoid unnecessary excessive chemotherapy. The present study indicated that single prognostic markers often provide inaccurate and incomplete prognostic indexes. Therefore, combined detection provides significant insight into an accurate prognosis. To confirm the accurate prognostic value of PTEN, p27 and Cyclin D1, a larger group of patients should be enrolled in future blinded, prospective studies.

## Figures and Tables

**Table I:** PTEN, p27 and Cyclin D1 expression in the control and CRC groups.

		PTEN		p27		Cyclin D1	
				
Group	Total	Positive, n (%)	P-value	Positive, n (%)	P-value	Positive, n (%)	P-value
Control	20	20 (100.00)	0.001	20 (100.00)	0.008	0 (0.0)	0.001
CRC	61	35 (57.38)		41 (67.21)		28 (45.90)	

PTEN, phosphatase and tensin homolog; CRC, colorectal cancer.

**Table II:** Correlation between expression of PTEN, p27 and Cyclin D1 and clinicopathological characteristics in 61 patients with CRC.

		PTEN		p27		Cyclin D1	
				
Variables	Total, n	Positive, n (%)	P-value	Positive, n (%)	P-value	Positive, n (%)	P-value
Age, years			0.979		0.796		0.768
≥60	37	23 (62.2)		26 (70.3)		20 (54.1)	
<60	24	12 (50.0)		15 (62.2)		8 (33.3)	
Gender			0.241		0.052		0.068
Male	38	24 (63.2)		29 (76.3)		14 (36.8)	
Female	23	11 (47.8)		12 (52.2)		14 (60.9)	
Tumor grade			0.001		0.004		0.207
Moderate	46	32 (69.6)		36 (78.3)		19 (41.3)	
Poor	15	3 (20.0)		5 (33.3)		9 (60.0)	
Tumor invasion			0.355		0.316		0.003
T3	30	19 (63.3)		22 (73.3)		8 (26.7)	
T4	31	16 (51.6)		19 (61.3)		20 (64.5)	
Lymph metastasis			0.001		0.001		0.011
N0	32	26 (81.3)		28 (87.5)		9 (28.1)	
N1	18	7 (38.9)		9 (50.0)		12 (66.7)	
N2	11	2 (18.2)		4 (36.4)		7 (63.6)	
Clinical stage			0.002		0.001		0.002
II	32	26 (81.3)		28 (87.5)		9 (28.1)	
III	29	9 (31.0)		13 (44.8)		19 (65.5)	

PTEN, phosphatase and tensin homolog; CRC, colorectal cancer.
